# White Matter Neuroplasticity: Motor Learning Activates the Internal Capsule and Reduces Hemodynamic Response Variability

**DOI:** 10.3389/fnhum.2020.509258

**Published:** 2020-10-26

**Authors:** Tory O. Frizzell, Lukas A. Grajauskas, Careesa C. Liu, Sujoy Ghosh Hajra, Xiaowei Song, Ryan C. N. D’Arcy

**Affiliations:** ^1^Simon Fraser University ImageTech Lab, Health Science and Innovation, Surrey Memorial Hospital, Fraser Health, Surrey, BC, Canada; ^2^Faculty of Applied Sciences, Simon Fraser University, Burnaby, BC, Canada; ^3^Cumming School of Medicine, University of Calgary, Calgary, AB, Canada; ^4^Department of Biomedical Physiology and Kinesiology, Simon Fraser University, Burnaby, BC, Canada; ^5^Rotman Research Institute, Baycrest Health Sciences, Toronto, ON, Canada; ^6^Flight Research Laboratory, National Research Council Canada, Ottawa, ON, Canada; ^7^Djavad Mowafaghian Centre for Brain Health, University of British Columbia, Vancouver, BC, Canada

**Keywords:** functional magnetic resonance imaging, white matter activation, neuroplasticity, motor learning, functional connectivity, internal capsule

## Abstract

Numerous studies have noted the importance of white matter changes in motor learning, but existing literature only focuses on structural and microstructural MRI changes, as there are limited tools available for *in vivo* investigations of white matter function. One method that has gained recent prominence is the application of blood oxygen level dependent (BOLD) fMRI to white matter, with high-field scanners now being able to better detect the smaller hemodynamic changes present in this tissue type compared to those in the gray matter. However, fMRI techniques have yet to be applied to investigations of neuroplastic change with motor learning in white matter. White matter function represents an unexplored component of neuroplasticity and is essential for gaining a complete understanding of learning-based changes occurring throughout the whole brain. Twelve healthy, right-handed participants completed fine motor and gross motor tasks with both hands, using an MRI compatible computer mouse. Using a crossover design along with a prior analysis approach to establish WM activation, participants received a baseline scan followed by 2 weeks of training, returning for a midpoint and endpoint scan. The motor tasks were designed to be selectively difficult for the left hand, leading to a training effect only in that condition. Analysis targeted the comparison and detection of training-associated right vs left hand changes. A statistically significant improvement in motor task score was only noted for the left-hand motor condition. A corresponding change in the temporal characteristics of the white matter hemodynamic response was shown within only the right corticospinal tract. The hemodynamic response exhibited a reduction in the dispersion characteristics after the training period. To our knowledge, this is the first report of MRI detectable functional neuroplasticity in white matter, suggesting that modifications in temporal characteristics of white matter hemodynamics may underlie functional neuroplasticity in this tissue.

## Introduction

White matter (WM) is a critically important tissue for brain function, making up almost 50% of the brain’s volume (Black, 2007), and providing connections between cortical areas in a manner vital for communication throughout neural networks (Bassett et al., 2011). Despite this, WM has been largely excluded from fMRI investigations. By extension, very little is known about the role of WM in brain network activation, and changes in functional activity with time. By focusing neuroplasticity investigations solely on GM areas of brain we restrict our understanding of the complex changes that underly processes such as learning and rehabilitation. Indeed, a focus on WM activation is critical as this has historically been a “blind spot” in functional MRI research (Grajauskas et al., 2019).

Though studies using blood oxygen level dependent (BOLD) signal have largely focused on gray matter (GM), improved MRI technology is allowing for the detection of the lower amplitude BOLD signals coming from WM (Gawryluk et al., 2014b). Early investigations into capturing WM BOLD signal found it detectable at high field strengths. Gawryluk et al. (2009, 2011) demonstrated WM fMRI signal at 4T by using an asymmetric spin echo (ASE) sequence to improve sensitivity to the comparatively smaller WM signal. Higher field strengths have an increased ability to detect WM BOLD activation (Mazerolle et al., 2013), and ASE sequences likewise have increased sensitivity (McWhinney et al., 2012). Gawryluk et al. (2014b), Grajauskas et al. (2019), and Li et al. (2019) provide reviews of the evidence in support of the biological basis of WM BOLD fMRI activation.

A great deal of the existing WM BOLD research has focused on the corpus callosum, as the large and isolated nature of the tract makes it ideal for investigations of WM activity (Gawryluk et al., 2009, 2014a; Mazerolle et al., 2013). However, there have also been numerous reports of detectable activation in the internal capsule, linked with motor functions such as swallowing (Mosier and Bereznaya, 2001) and finger tapping tasks (Mazerolle et al., 2010; Gawryluk et al., 2011). Similar to the corpus callosum results (Mazerolle et al., 2010), WM activation in the internal capsule has been integrated with diffusion tensor imaging (DTI) results and the larger GM activation network.

Following the initial technical research, WM BOLD signal was subsequently detected in a number of resting state and task-based fMRI studies (Marussich et al., 2017; Peer et al., 2017; Wu et al., 2017; Zhou et al., 2018) at conventional field strengths. The successful detection of WM BOLD at conventional field strengths and standard sequences improved the accessibility and adoptability within the field of research.

More recently, advanced analysis has investigated the unique hemodynamics of white matter, showing that there are different patterns of blood flow within this tissue type. Courtemanche et al. (2018) demonstrated that sensitivity to WM BOLD signals could be improved by using a WM specific hemodynamic response function (HRF). The hemodynamic response in the corpus callosum has been shown to have a delayed onset slope and delayed peak time when compared to GM (Courtemanche et al., 2018). Li et al. (2019) further confirmed this and found that the hemodynamic profile of WM may differ considerably throughout the brain (Li et al., 2019). This is a contributing factor to the lack of detected WM activation in the literature, as the use of hemodynamic response functions (HRFs) modeled on the properties of GM may not properly reflect the WM signal (Grajauskas et al., 2019).

Though WM is an active functional tissue, investigation into the role of WM in neuroplasticity has historically been limited to structural and microstructural measures. DTI is a leading modality, and though it only offers structural information, it is the foundation of WM neuroplasticity quantification. Specifically, fractional anisotropy (FA) and mean diffusivity (MD) have been used as key biomarkers of WM structure. Numerous studies have demonstrated a measurable change in FA and MD during longitudinal monitoring of visual and motor training tasks (Scholz et al., 2009; Takeuchi et al., 2010; Taubert et al., 2010; Hofstetter et al., 2013; Wang et al., 2014; Yotsumoto et al., 2014). Cross sectional investigations corroborate this link between WM structure and function. Proficiency in skills such as music, dance, and fine motor tasks have been linked with population difference in FA (Bengtsson et al., 2005; Johansen-Berg et al., 2007; Imfeld et al., 2009; Hänggi et al., 2010; Steele et al., 2013). Significant changes in WM tract myelination, another key biomarker of WM structure, have also been shown in visuo-motor training tasks (Lakhani et al., 2016).

More recently, a 4-week motor training activity was shown to increase FA in several white matter tracts associated with motor activity. An increase in FA within the right CST, along a tract seeded from areas activated by the tapping task, was found (Reid et al., 2017). This task showed a significantly lateralized difference in motor training across several modalities between participants non-dominant and dominant hands. The evidence of motor learning-based changes in fMRI (Sale et al., 2017) and WM structure (Reid et al., 2017) provided the model for our experimental design in order to best elicit evidence of WM functional neuroplasticity.

Given the established role of WM in motor learning, the aim of this study was to apply functional measures of motor learning and plasticity. The first objective was to extend previous findings of WM activation in the internal capsule, showing lateralized activation based on the hand used during the task. Given the novel experimental paradigm, an established analysis approach was used to first establish WM activation. The second objective was to show WM activation changes consistent with a behavioral motor learning effect. Building on the established differences in WM hemodynamic properties, we included metrics to assess HRF changes beyond traditional measures of BOLD amplitude and extent. This allowed a more complete investigation into functional WM changes during neuroplasticity.

Hypothesis 1 predicted that WM activation would be detectable in the contralateral internal capsule for both non-dominant and dominant hands. Hypothesis 2 predicted reduced WM HRF variability as a function of neuroplasticity, particularly in the non-dominant hand. The research employed a repeat measures crossover design that included three longitudinal scan sessions (baseline, midpoint, and endpoint).

## Materials and Methods

### Participants

Twelve healthy, right-handed participants were recruited (7 females). The mean age of the participants was 25.8 ± 3.7 years. Right hand dominance was confirmed using the Edinburgh Handedness Inventory (Oldfield, 1971). Subjects did not report psychiatric or neurological illness, or recent concussions. Written informed consent was obtained from all subjects.

### Experimental Design

A 2-week motor training experiment was developed, similar in design as Reid et al. (2017) and Sale et al. (2017). The motor training block task paradigm was developed in PsychToolbox3 (Brainard, 1997; Pelli, 1997), a package of MATLAB (R2018b, The MathWorks, Inc.). Participants used an MRI compatible mouse (Nata Technologies, Coquitlam B.C.) to guide a cursor through a marked trail displayed on the monitor. Participants were instructed to minimize errors and maximize distance traveled. Performance metrics were recorded based on speed and accuracy. The task difficulty was designed to emphasize the difference in dominant and non-dominant hand proficiency.

Participants received a baseline scan during which a fine motor learning task was completed once with each hand, counterbalanced with a matched gross motor comparison task (results not included for brevity). The fine motor learning task involved accurate, rapid tracing of randomize trail mazes, with performance scores incorporating distance and error-rate. All scans were 6 min long with seven on-blocks of 24 s each during which a unique trail was displayed. These on blocks were interleaved with jittered rest blocks averaging 24 s. Each scan began and ended with a rest block. The trails were designed to be too complex to be completed in a single 24 s block. The gross motor training task followed the same block structure and visual display as the fine motor task, but simply had the participants move the mouse at random, with no accuracy metrics.

Following the baseline scan, participants completed 2 weeks of daily “at-home” task training with both non-dominant and dominant hands. During the training period each participant underwent a midpoint scan (after 1 week) and an endpoint scan following the same scanning protocol as baseline.

### MRI Acquisition

MRI scans were acquired from using a 3 Tesla Philips Ingenia CX MRI scanner with a 32-channel dStream head coil. Functional MRI images were acquired using an FFE single-shot GRE-EPI sequence. The acquisition parameters were as follows: TR = 2,000 ms, TE = 30 ms, and flip angle = 90°. Data were collected with an in-plane resolution of 3 × 3 mm and slice thickness 3 mm with matrix dimensions of 80 × 80 voxels and 36 slices.

High-resolution T1-weighted images were collected at each session for co-registration of functional images. The acquisition parameters were as follows: TR = 8.2 ms, TE = 3.7 ms, and flip angle = 8°. Data were collected with an in-plane resolution of 1 × 1 mm and slice thickness of 1 mm with matrix dimensions of 288 × 288 voxels and 213 slices.

### MRI Analysis

Data were analyzed using FMRIB Software Library 6.0.0 (FSL) (Jenkinson et al., 2012). Pre-statistical processing was completed using FEAT (6.0), and brain extraction was completed using BET. Images were motion corrected using MCFLIRT, spatially smoothed using a Gaussian kernel of FWHM 5 mm, and high pass filtered with a cutoff of 100 s. Functional images were registered to T1 weighted anatomical images using FLIRT, and to MNI152 standard space using FNIRT.

To test the hypotheses, analysis was targeted specifically to WM functional activity as the changes in GM BOLD signal will be the subject of future analyses. To this end, predictors were constructed in a manner based on the work of Courtemanche et al. (2018), which compared the use of a conventional GLM to a model that included HRF basis sets generated in FLOBS. This created an HRF designed to better reflect white matter hemodynamics. Courtemanche et al. (2018) showed increased sensitivity to WM activation using the WM HRF, and as such we have followed these procedures in our current work. This method used a linear optimized basis set for the HRF convolution generated in the FSL toolkit “FMRIB’s Linear Optimal Basis Sets” (FLOBS) (Woolrich et al., 2004). The parameters used follow the procedures laid out in Courtemanche et al. (2018) and were inputted as follows: M1 = 0–4 s, M2 = 3–12 s, M3 = 3–5 s, M4 = 3–5 s, c = 0–0.3, Bases = 3. These parameters specify a range of example HRFs, with delayed onset slope and a reduced initial overshoot, to better reflect WM hemodynamics (Tae et al., 2014). From this set of HRFs, FLOBS uses principal component analysis to generate an “optimal basis set” which maximally spans the specified space. Further information on the proofs underlying the FLOBS toolkit can be found in the FSL technical documentation, or in Woolrich et al. (2004).

Three bases were generated; an “HRF-like” curve, a “latency derivative” that modeled the temporal lag of the HRF, and a “dispersion derivative” that modeled differences in width of the HRF. The bases were convolved with the stimulus time series to determine group differences of both HRF amplitude and associated temporal characteristics (Ward et al., 2008).

Statistical analyses were computed using a generalized linear model for generating cluster-based contrasts corrected for multiple-comparisons. Individual level z-statistics were generated from task vs. rest contrast using significance thresholds of *z* > 2.5 (*p* < 0.05, corrected).

Group level statistical analyses were computed using FSL’s FEAT function with FLAME mixed effects. Timepoint contrast for group level analyses compared baseline and endpoint with significance thresholds of (*p* < 0.05, FWE) for each of the HRF bases.

Regions of interest masks of white matter were generated from the Harvard-Oxford subcortical structure atlas using conservative thresholds (50%) to reduce the chance of partial volume effects. Masks of gray matter areas associated with motor learning in tracing tasks were generated from the Jülich Histological Atlas and the MNI Structural atlas using typical thresholds (20%).

The timeseries BOLD data within the right internal capsule ROI were analyzed during the left-hand task. The BOLD signal was averaged across voxels within the ROI and the timeseries data were windowed around the stimulus presentation times. Subsequently, the timeseries mean were subtracted from the BOLD response. Group averaged ROI BOLD intensity were plotted with respect to stimulus onset times for the baseline and endpoint data. Additionally, the group level standard deviation in BOLD signal was computed and plotted at the baseline and endpoint.

### Behavioral Analysis

A two way repeated measures ANOVA was conducted to investigate the effect of hand and scan timepoint on the behavioral scores. *Post hoc t*-tests (pairwise, two-tailed) were run to interrogate the timepoint contrasts of the behavioral results, looking at the difference between baseline and endpoint scan for each hand. The significance level was set using Bonferroni adjusted *p* < 0.025 across the two *t*-tests (α = 0.05/2).

## Results

### Behavioral Performance

The two way repeated measures ANOVA confirmed a significant effect of hand and scan timepoint on the behavioral scores. The *post hoc t*-tests (pairwise, two-tailed) revealed a significant difference for the left-hand tracing scores between baseline and endpoint, (*p* < 0.005, α = 0.025). After Bonferroni correction for multiple comparisons all results remained significant. There was no significant difference for the right-hand tracing scores between baseline and endpoint, (*p* = 0.099).

### Individual Timepoint WM fMRI

**FIGURE 1:**
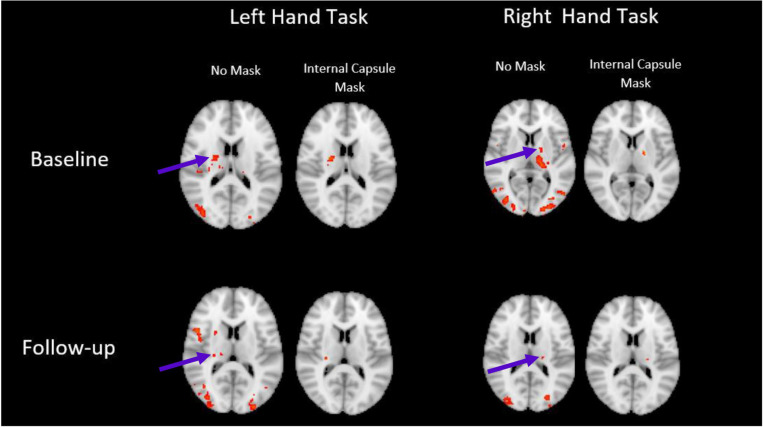
WM activation revealed in the internal capsule. Activation is lateralized as expected, and still present when conservatively thresholded masks are applied. Images displayed in radiological convention, right side of brain on left side of image. (*z* > 4, *P* < 0.05, FWE corrected).

The analysis of fMRI data was collapsed across groups and analyzed as a baseline vs. endpoint contrast, focusing on our non-dominant/dominant hand training comparison. Local maxima of activation were detected within the internal capsule in a lateralized manner at the group level for both baseline and endpoint with typical thresholds of *z* > 2.5. In order to isolate the white matter local maxima, data were re-thresholded at *z* > 4 (*p* < 0.05, FWE), revealing several peaks of activation within white matter areas. Cluster maps were then masked using the internal capsule ROI as seen in Figure 1. Resulting internal capsule activation is summarized in Table 1.

**TABLE 1:** Voxels showing activation within the internal capsule at each timepoint (*z* > 4, *P* < 0.05, FWE corrected, clusters of 10 or more voxels reported).

	ROI	#voxels	Z-max	X (mm)	Y (mm)	Z (mm)
Left task baseline	Right internal capsule	185	5.17	28	-20	6
		12	4.77	10	−26	−12
Left task endpoint	Right internal capsule	56	4.80	20	−20	6
		18	4.71	26	−24	48
Right task baseline	Left internal capsule	20	4.55	−18	−6	4
Right task endpoint	Left internal capsule	22	4.68	−26	−28	50
		10	4.32	−20	−22	10

**Z-max is the peak z score within the ROI, with location specified in MNI coordinates.**

### Timepoint Comparisons

While WM activation was detected across timepoints, a baseline vs. endpoint comparison revealed no detectable changes, even at low thresholds.

A major objective of this research was to investigate the temporal changes of the hemodynamic response in white matter as a functional of neuroplasticity. The subsequent analysis investigated the changes in temporal characteristics of the HRF pre and post training. Timepoint comparisons of the latency derivative revealed no significant findings in any condition.

**FIGURE 2:**
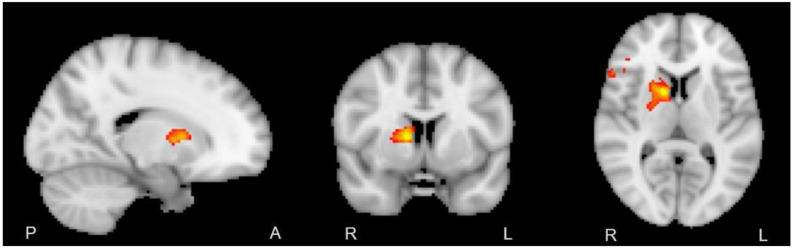
Right internal capsule showing greater HRF dispersion at group level, baseline > endpoint (*z* > 2.5, *p* < 0.05, FWE corrected). No mask applied.

**FIGURE 3:**
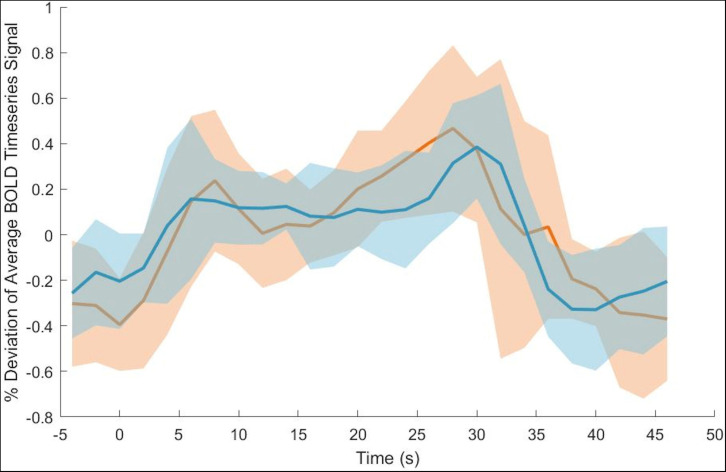
Average across ROI voxels with subtracted mean BOLD signal intensity for baseline (orange) and endpoint (blue). Group level standard deviation plotted around timepoint BOLD signal intensity means. Time at 0 s represents the stimulus onset time. Note that the standard deviation is greater at baseline than endpoint.

However, group level Baseline > Endpoint comparisons of the dispersion derivative revealed a significant cluster of voxels within the internal capsule for the non-dominant hand tracing condition (Figure 2). Information for the significant cluster is summarized in Table 2. This shows a significant reduction in variance of the HRF in WM motor related tracts after the training period. The change in dispersion is aligned well with the structure of the internal capsule, as can be noted in Figure 2 (peak cluster voxel 82.7% right cerebral white matter, Harvard-Oxford Subcortical Structure Atlas). Figure 3 presents a comparison between the group block averaged time series data at baseline and endpoint for voxels within the internal capsule ROI. No other conditions revealed significant changes. The reverse contrast revealed no significant results for any condition.

**TABLE 2:** Voxels displaying positive parameter estimates for the timepoint 1 > timepoint 3 contrast of the dispersion derivative (*z* > 2.5, *P* < 0.05, FWE corrected).

Condition	ROI	# Voxels	Z-max	X (mm)	Y (mm)	Z (mm)
Left task	Right internal capsule	180	3.51	14	8	8

**Z-max is the peak z score within the ROI, with location specified in MNI coordinates.**

To better visualize the changes occurring in the WM HRF dispersion derivative Figure 3 plots the group BOLD signal averages and standard deviation.

## Discussion

The results supported Hypothesis 1, with WM activation detected in the contralateral internal capsule for both non-dominant and dominant hands. Examination of WM HRF results also supported Hypothesis 2, with reduced WM HRF dispersion as a function of neuroplasticity in the non-dominant hand.

Task fMRI revealed clusters of activation with local maxima within the internal capsule, in a manner lateralized as expected (Figure 1; right internal capsule activity during the left-hand task, left internal capsule activity during the right-hand task). A conservative white matter mask of the internal capsule and corticospinal tract was used to verify that the clusters were not the result of partial volume effects. Additionally, in Figure 1 it can be noted that even without masking, the detected activation occurs in an area without adjacent gray matter activation, further addressing partial volume effects.

The use of a WM specific HRF was key for increasing sensitivity to WM functional activation at conventional MRI field strengths. Our results align well with previous reports of WM activation in the internal capsule (Gawryluk et al., 2011), with similar lateralization and localization of activation. However, previous studies were completed in a 4T scanner, using a specialized ASE sequence, optimized for WM detection. Higher field strengths have a notable impact on the ability to detect WM BOLD activation (Mazerolle et al., 2013) and ASE sequences have improved sensitivity compared to the traditional GRE-EPI sequences (McWhinney et al., 2012). The strong agreement between our current results and previously published reports of activation in the internal capsule (Maldjian et al., 1999; Mosier et al., 1999; Gawryluk et al., 2011) supports the conclusion that a WM specific HRF model is critical for obtaining a holistic representation of brain function (Li et al., 2019).

The analysis into adaptation of HRF profile over time revealed significant reduction in the dispersion (Figure 3). The white matter hemodynamic changes selectively occurred in the contralateral internal capsule for the non-dominant hand only. As seen in Figure 2 this cluster aligns well with white matter motor related tracts, even at conventional thresholds with no masking. This finding paralleled the behavioral results where only the non-dominant hand showed significant learning effects.

It can be noted that even without masking, the pattern of maximum HRF dispersion change occurs over the anatomy of the internal capsule, with a local maximum occurring at the genu, and activation spreading backwards along the posterior limb. Though motor signals from the corticospinal tract are traditionally thought to travel through the posterior limb of the internal capsule, more recent research has debated the exact localization of these representations (Duerden et al., 2011; Qian and Tan, 2017) and the function of the genu (Yim et al., 2013). Evidence from non-human primates using histopathology and retrograde traces from motor areas showed that arm representations are distributed throughout the internal capsule in an overlapping manner, with fibers from the supplementary motor area passing through the genu (Morecraft et al., 2002).

In future, the investigation into the temporal characteristics of the WM HRF represents a novel detection of functional neuroplasticity in WM. Variation in HRFs is typically quantified using the dispersion derivative (Storti et al., 2013). HRF profiles have been shown to be extremely variable in white matter, contrary to the consistent HRF throughout gray matter (Li et al., 2019), and thus warrant further investigation as they relate to neuroplasticity. Given the variability of HRF shapes within separate WM areas, modeling the temporal and dispersion derivatives at group level was essential for the detection of WM functional changes (Courtemanche et al., 2018; Li et al., 2019). In particular, direct examination of spatial changes in HRFs through novel methods like functional correlation tensors (FCT; Ding et al., 2013) will be important to evaluate the detectable extent of BOLD fMRI activity within the larger WM tract. Given that specific GM HRF assumptions around features and analysis approaches may not generalize to WM HRFs, the continued work to narrow down WM-specific features and analysis approach may also be more complex in light of the spatially distributed nature of the activation across WM tracts. Multimodal integration with DTI and other methods will therefore be critical.

The recorded changes in WM function are intrinsically linked to changes in structure. Reid et al. (2017) and Sale et al. (2017) found increases in fractional anisotropy, seeded by sensorimotor cortex fMRI activation, throughout the right corticospinal tract after a similar motor training paradigm. We expect that similar changes in the structure of the internal capsule as a result of motor training are responsible for the observed changes in WM hemodynamic response in this work. A better understanding of brain changes in terms of both structure and function is essential for a comprehensive picture of the impact of motor training and rehabilitation.

While fMRI lacks the ability to draw direct inferences about the underlying cellular or hemodynamic processes at play, there are a few possible physiological mechanisms that may be driving this change. We postulate that the most probable microstructural change underlying our results is an increase WM tract myelination (Sampaio-Baptista and Johansen-Berg, 2017). This phenomenon, that has been detected in motor related WM tracts during training (Lakhani et al., 2016; Reid et al., 2017; Sale et al., 2017), may drive an increase in neural efficiency. Increased myelination within the white matter tracts improves efficient propagation of action potentials, due to a reduced ion flux during each action potential propagation and thus reduced work to re-establish the resting membrane potential (Neishabouri and Faisal, 2011). More efficient action potentials reduce the rate in which energy stores in the neurons are depleted, thus changing their metabolic demands and the resulting hemodynamic response. Underlying factors, such as these, understandably contribute to variation in the HRF profile.

Despite the reports of structural and microstructural changes throughout the corticospinal tract no activation was detected in the corticospinal tract above the internal capsule. This too reflects several previous reports of isolated activation in this region (Maldjian et al., 1999; Mosier et al., 1999; Gawryluk et al., 2011). WM exhibits a high level of anisotropy compared to GM, meaning that the neural tissue and supporting vasculature are largely oriented in a single direction. Anisotropic tissue oriented parallel to the main magnetic field has a reduced ability to produce a detectable BOLD signal (Hernández-Torres et al., 2017; Doucette et al., 2019). The majority of the corticospinal tract is one such anisotropic tract, which aligns parallel to the main magnetic field. The internal capsule, however, exhibits greater isotropy of the fibers compared to the rest of the corticospinal tract which may offer an explanation for the bounded BOLD signal detection in this region.

While the current work supports the idea that WM activation can be used to track neuroplasticity, there are a few limitations to interpretation. First, we employed a basic block design, that allowed for higher amplitude hemodynamic responses, helping to compensate for the smaller hemodynamic changes seen in white matter. A jittered interstimulus interval was employed to eliminate long latency hemodynamic effects and allow for the interrogation of latency and dispersion effects. However, employing an event related design in future work will allow for more granular inferences about short latency effects. This work also used a generalized linear model to investigate significant temporal changes between time points. This allowed us to highlight areas where the most change occurred. However, sampling HRFs throughout the white matter and comparing them could allow for comparison within and between white matter tracts. The sample size of this investigation is small and represents an important consideration for statistical power. Future analyses would benefit from a greater participant number, particularly for detecting small signal changes inherent to WM BOLD.

Future work will use a technique called functional correlation tensors (Ding et al., 2013) that can be used to analyze local synchronicity in hemodynamic fluctuations. Functional correlation tensors will be used to further investigate the reduced variability in hemodynamic response throughout the whole brain. Future investigations will also explore co-localizing functional results with DTI to relate WM structural changes with WM BOLD neuroplasticity. Corresponding structural or microstructural information is needed to further understand the underlying mechanisms of WM fMRI activation.

This work has determined that a 2-week motor training task can elicit detectable changes in WM BOLD signal. The temporal characteristics of the WM HRF can be altered as a result of training; manifesting as a reduction in the dispersion of the hemodynamic response. This work has highlighted importance of the temporal characteristics of functional activity, particularly for longitudinal analysis of WM activation. This represents an important new direction for future analyses of both white matter neuroplasticity, as well as analysis of BOLD activation in white matter.

## Data Availability Statement

The raw data supporting the conclusions of this article will be made available by the authors, without undue reservation, to any qualified researcher.

## Ethics Statement

The studies involving human participants were reviewed and approved by the Simon Fraser University Research Ethics Board. The patients/participants provided their written informed consent to participate in this study.

## Author Contributions

LG and TF contributed equally as primary authors with data acquisition, analysis and manuscript drafting. CL and SG provided support in study design and manuscript editing. XS and RD’A assisted in editing the manuscript and acted as lab PI. All authors contributed to the article and approved the submitted version.

## Conflict of Interest

The authors declare that the research was conducted in the absence of any commercial or financial relationships that could be construed as a potential conflict of interest.
